# Evaluation of Cerebrospinal Fluid **α**‐Synuclein Seed Amplification Assay in Progressive Supranuclear Palsy and Corticobasal Syndrome

**DOI:** 10.1002/mds.30019

**Published:** 2024-09-20

**Authors:** David P. Vaughan, Riona Fumi, Marte Theilmann Jensen, Megan Hodgson, Tatiana Georgiades, Lesley Wu, Danielle Lux, Ruth Obrocki, Jennifer Lamoureux, Olaf Ansorge, Kieren S.J. Allinson, Thomas T. Warner, Zane Jaunmuktane, Anjum Misbahuddin, P. Nigel Leigh, Boyd C.P. Ghosh, Kailash P. Bhatia, Alistair Church, Christopher Kobylecki, Michele T.M. Hu, James B. Rowe, Cornelis Blauwendraat, Huw R. Morris, Edwin Jabbari

**Affiliations:** ^1^ Department of Clinical and Movement Neurosciences UCL Queen Square Institute of Neurology London United Kingdom; ^2^ Movement Disorders Centre, UCL Queen Square Institute of Neurology London United Kingdom; ^3^ Amprion Inc. San Francisco California USA; ^4^ Nuffield Department of Clinical Neurosciences University of Oxford Oxford United Kingdom; ^5^ Department of Clinical Neurosciences Cambridge University Hospitals NHS Trust and MRC Cognition and Brain Sciences Unit, University of Cambridge Cambridge United Kingdom; ^6^ Reta Lila Weston Institute, UCL Queen Square Institute of Neurology London United Kingdom; ^7^ Queen Square Brain Bank for Neurological Disorders, UCL Queen Square Institute of Neurology London United Kingdom; ^8^ Department of Neurology Queen's Hospital Romford United Kingdom; ^9^ Department of Neuroscience Brighton and Sussex Medical School Brighton United Kingdom; ^10^ Wessex Neurological Centre, University Hospitals Southampton NHS Foundation Trust Southampton United Kingdom; ^11^ Department of Neurology Royal Gwent Hospital Newport United Kingdom; ^12^ Department of Neurology Northern Care Alliance NHS Foundation Trust, Manchester Academic Health Science Centre, University of Manchester Manchester United Kingdom; ^13^ Laboratory of Neurogenetics, National Institute on Aging, National Institutes of Health Bethesda Maryland USA; ^14^ Center for Alzheimer's and Related Dementias, National Institute on Aging and National Institute of Neurological Disorders and Stroke, National Institutes of Health Bethesda Maryland USA

**Keywords:** synucleinopathies, tauopathies, biomarker, diagnosis, neuropathology

## Abstract

**Background:**

Seed amplification assay (SAA) testing has been developed as a biomarker for the diagnosis of α‐synuclein‐related neurodegenerative disorders.

**Objective:**

The objective of this study was to assess the rate of α‐synuclein SAA positivity in progressive supranuclear palsy (PSP) and corticobasal syndrome (CBS) and to analyze clinical and pathological features of SAA‐positive and ‐negative cases.

**Methods:**

A total of 96 cerebrospinal fluid samples from clinically diagnosed PSP (n = 59) and CBS (n = 37) cases were analyzed using α‐synuclein SAA.

**Results:**

Six of 59 (10.2%) PSP cases were α‐synuclein SAA positive, including one case who was MSA‐type positive. An exploratory analysis showed that PSP cases who were Parkinson's disease–type positive were older and had a shorter disease duration compared with SAA‐negative cases. In contrast, 11 of 37 (29.7%) CBS cases were α‐synuclein SAA positive, including two cases who were MSA‐type positive.

**Conclusions:**

Our results suggest that α‐synuclein seeds can be detected in PSP and CBS using a cerebrospinal fluid α‐synuclein SAA, and in PSP this may impact on clinical course.

## Graphical abstract

α‐Synuclein seed amplification assay (SAA)‐positive results were found in 6 of 59 (10.2%) progressive supranuclear palsy (PSP) and 11 of 37 (29.7%) corticobasal syndrome (CBS) cerebrospinal fluid (CSF) samples, potentially representing Lewy body copathology. An exploratory analysis showed that PSP cases who were Parkinson's disease–type SAA positive were older and had a shorter disease duration compared with SAA‐negative cases.

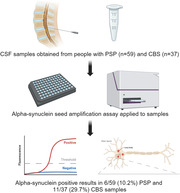

The neuropathology of progressive supranuclear palsy (PSP) involves accumulation of neuronal and glial hyperphosphorylated 4‐repeat tau (4RT) pathology with associated neurodegeneration,^1^ and a clinical diagnosis of PSP is strongly predictive of underlying PSP pathology at postmortem.^2^ In contrast, the underlying neuropathology of corticobasal syndrome (CBS) is diverse, including corticobasal degeneration (CBD; also a 4‐repeat tauopathy), PSP, and Alzheimer's disease (AD).^3^, ^4^

Despite recent updates to clinical diagnostic criteria for PSP and CBS, which acknowledge the clinical heterogeneity of these conditions,^5^, ^6^ the diagnosis of PSP and CBS in the early stages of symptomatic disease is challenging in the absence of objective disease‐specific diagnostic biomarkers. This is due to clinical overlap with other parkinsonian disorders, including Parkinson's disease (PD) and multiple system atrophy (MSA), which are characterized by the accumulation of α‐synuclein neuropathology.

There is increasing evidence that α‐synuclein seed amplification assays (SAAs) can reliably differentiate people with PD from healthy control subjects with high sensitivity and specificity, and may even be able to differentiate PD from other synucleinopathies, including MSA.^7^ In addition, the assay results show good interlaboratory concordance,^8^ can be applied to a range of biofluids (including cerebrospinal fluid [CSF], blood, skin, and saliva) with variable sensitivities and specificities, and show a high rate of positivity in the prodromal phase of synucleinopathies.^7^ Consequently, there is a drive to move away from a clinical diagnosis of PD toward a biological definition of α‐synuclein disease based on SAA positivity to enable clinical trials at early disease stages.^9^, ^10^ Furthermore, there are ongoing efforts to develop effective 4RT SAAs.^11^

In this study, we evaluate the α‐synuclein SAA in a cohort of PSP and CBS CSF samples with matched clinical and postmortem data.

## Subjects and Methods

### Participant Recruitment and Diagnosis

Written informed consent was obtained from people with PSP and CBS who were recruited to the PROSPECT‐UK study between September 1, 2015, and November 1, 2023 (Supporting Information).

### Clinical, Biomarker, and Neuropathological Data Collection

We obtained clinical data and completed core clinical assessments at baseline, and where possible these were repeated after 6, 12, 24, and 36 months of follow‐up (Supporting Information).

Lumbar puncture was an optional study assessment to obtain baseline CSF for biomarker analyses. CBS cases were stratified into CBS‐Alzheimer's (CBS‐AD) and CBS‐non‐Alzheimer's (CBS‐non‐AD) groups based on the presence of CSF or positron emission tomography imaging biomarkers of AD pathology (Supporting Information). In those undergoing postmortem, the primary neuropathological diagnosis was noted along with the presence or absence of Lewy body copathology and associated Braak stage.

Baseline CSF samples from participants were applied to the Amprion α‐synuclein SAA (Supporting Information). After a total incubation time of 20 hours, the maximum fluorescence for each well is determined and an algorithm applied to the triplicate determinations for each sample for result classification (positive, negative, or indeterminate). Kinetic parameters of the aggregation curve were used by Amprion to assign positive results as “PD‐type” or “MSA‐type” as described previously.^12^

### Statistical Analysis

Group (PSP vs. CBS) and subgroup (SAA^+^ vs. SAA^−^) comparisons were performed with Fisher's exact test for categorical variables and binary logistic regression, adjusting for sex, age, and disease duration at assessment, for comparisons of continuous variables. Statistical significance was set at *P <* 0.05. All statistical analyses were performed in GraphPad Prism 9.1.1 (San Diego, CA, USA).

## Results

A total of 96 participants from the PROSPECT‐UK study who had undergone a baseline lumbar puncture and clinical assessment were included in this study consisting of 59 clinically diagnosed PSP participants and 37 clinically diagnosed CBS participants (CBS‐AD, n = 13; CBS‐non‐AD, n = 18; CBS‐unknown, n = 6). The baseline clinical characteristics of the PSP and CBS groups were largely comparable and are summarized in Table 1. In those undergoing postmortem analysis (n = 12), an antemortem clinical diagnosis of PSP was always predictive of underlying 4RT neuropathology (five PSP, one CBD), in contrast with CBS (two PSP, two CBD, one MSA, one AD).

**TABLE 1:** Clinical and biomarker characteristics of PSP and CBS groups

Characteristics	PSP (n = 59)	CBS (n = 37)
Sex, % male	58%	30%*
CSF α‐synuclein SAA status, n		
Negative	52	26
PD‐type	5	9
MSA‐type	1	2
Indeterminate	1	
CSF AD biomarker status, n		
CBS‐AD		13
CBS‐non‐AD		18
CBS‐unknown		6
Clinical subtype at baseline assessment, n		
PSP‐RS	33	
PSP‐P	11	
PSP‐PGF	6	
PSP/CBS	5	
PSP‐SL	2	
PSP‐F	2	
Primary pathological diagnosis in participants undergoing postmortem, n		
PSP	5	2
CBD	1	2
MSA		1
AD		1
Mean age at symptom onset, y (SD)^a^	65.0 (6.9)	63.5 (7.5)
Mean age at baseline assessment, y (SD)^a^	69.0 (6.8)	68.2 (7.5)
Mean disease duration at baseline assessment, y (SD)^a^	4.0 (2.0)	4.7 (2.1)
Mean PSPRS score at baseline assessment (SD)^b^	33.7 (15.0)	31.0 (12.0)
Mean MDS‐UPDRS III score at baseline assessment (SD)^b^	36.3 (14.5)	43.9 (14.0)**
Mean MoCA score at baseline assessment (SD)^b^	22.2 (4.8)	19.6 (5.8)
Median SEADL score at baseline assessment (range)^b^	60 (10–90)	50 (20–90)
Participants undergoing assessment at two or more time points (n)	34	17
Mean latency between baseline and final assessments, y (SD)	1.6 (0.9)	1.7 (0.7)
Participants deceased at censoring (n)	28	19
Mean disease duration from symptom onset to death, y (SD)^a^	7.0 (3.0)	8.2 (3.2)

* *P* < 0.05 vs. PSP group using Fisher's exact test.

** *P* < 0.05 vs. PSP group using binary logistic regression that adjusted for sex, age, and disease duration at assessment. All other PSP vs. CBS group comparisons of continuous variables did not reach statistical significance (*P* > 0.05) using binary logistic regression that was both unadjusted^a^ and adjusted^b^ for covariates.

Abbreviations: PSP, progressive supranuclear palsy; CBS, corticobasal syndrome; CSF, cerebrospinal fluid; SAA, seed amplification assay; PD, Parkinson's disease; MSA, multiple system atrophy; AD, Alzheimer's disease; CBS‐AD, corticobasal syndrome‐Alzheimer's; CBS‐non‐AD, corticobasal syndrome‐non‐Alzheimer's; PSP‐RS, PSP‐Richardson's syndrome; PSP‐P, PSP‐parkinsonism; PSP‐PGF, PSP‐progressive gait freezing; PSP/CBS, PSP/CBS overlap; PSP‐SL, PSP‐speech and language disorder; PSP‐F, PSP‐frontal; CBD, corticobasal degeneration; SD, standard deviation; PSPRS, PSP rating scale; MDS‐UPDRS III, Movement Disorder Society–Unified Parkinson's Disease Rating Scale Part III; MoCA, Montreal Cognitive Assessment; SEADL, Schwab and England activities of daily living scale.

Six of 59 (10.2%) PSP participants were α‐synuclein SAA positive, including one MSA‐type positive participant who had probable PSP‐Richardson's syndrome and an absence of hyposmia, tremor, postural light‐headedness or visual hallucinations. One participant with probable PSP‐Richardson's syndrome had an indeterminate result and was excluded from subsequent analyses. In contrast, 11 of 37 (29.7%) CBS participants were α‐synuclein SAA positive, including two participants with probable CBS who were MSA‐type positive. One of these participants had an absence of synucleinopathy clinical features, whereas the other participant reported tremor and postural light‐headedness and went on to have postmortem confirmation of MSA as the primary pathological diagnosis.

Of the total cohort of postmortem cases, only one case (AD pathological diagnosis) was PD‐type SAA positive, and this case had Braak stage 1 Lewy body copathology. In the remaining postmortem cases, one pathologically diagnosed PSP case had Braak stage 1 Lewy body copathology but was PD‐type SAA negative. Therefore, in our pathologically confirmed cases, the sensitivity of the assay to detect primary α‐synuclein pathology or Lewy body copathology was 67% (2/3 positive cases), whereas the specificity was 100% (9/9 negative cases).

We then divided the PSP and CBS groups into PD‐type SAA‐positive and ‐negative groups. Although underpowered to detect statistical significance, this exploratory analysis showed a trend toward SAA positive PSP participants being older, more impaired in motor, cognitive, and functional scales, and having a more aggressive rate of progression compared with SAA‐negative PSP participants, as evidenced by a shorter average disease duration in deceased cases (5.8 vs. 7.2 years) (Table 2). In those undergoing serial clinical assessment, change in diagnosis in the PSP group was rare (2/33, 6.1%), and the rates of synucleinopathy clinical features were similar in SAA‐positive and ‐negative groups with the exception of hyposmia, which was more common in the SAA‐positive group. In contrast, SAA‐positive versus ‐negative clinical differences in the CBS group were less marked, although this was in the context of a higher overall rate of change in diagnosis (2/16, 12.5%) and long disease duration in SAA‐positive cases who were still alive at the point of censoring. Notably, a higher proportion of CBS SAA‐positive cases were classified as being AD biomarker positive (6/9 cases) compared with CBS SAA‐negative cases (7/26 cases); however, the CBS SAA‐negative group also included six cases with unknown AD biomarker status.

**TABLE 2:** PD‐type SAA status in PSP and CBS groups

PD‐Type SAA Status	PSP SAA Positive (n = 5)	PSP SAA Negative (n = 52)
PSP subtype (n)		
PSP‐RS	2	29
PSP‐SL	2	
PSP‐P	1	10
PSP‐PGF		6
PSP/CBS		5
PSP‐F		2
Diagnostic criteria certainty		
Probable criteria	3/5 cases	44/52 cases
Possible criteria	2/5 cases	8/52 cases
Change in clinical diagnosis	1/4 cases → IDT	1/29 cases → PD
Age at symptom onset (range)	71.7 (67–77) y	64.4 (48–77) y
Average age at baseline visit (range)	75.1 (70–80) y	68.4 (48–80) y
Disease severity		
PSPRS, average (SD)	38.6 (14.8)	33.7 (15.1)
MDS‐UPDRS III, average (SD)	42.0 (16.8)	36.1 (14.3)
SEADL^a^	40/100 (20–90)	60/100 (10–90)
MoCA, average (SD)	17.8 (4.1)	22.6 (4.7)
Disease duration in deceased cases, average (range)	5.8 (3.0–8.7) y	7.2 (3.3–16.8) y
Disease duration in alive cases, average (range)	6.3 (4.2–10.0) y	5.6 (2.4–12.4) y
Primary pathological diagnosis	N/A	PSP (n = 5); CBD (n = 1)
LB copathology, Braak stage	N/A	0/6 cases
Postural light‐headedness	3/4 cases (75%)	22/43 cases (51.2%)
Visual hallucinations	0/5 cases (0%)	5/49 cases (10.2%)
Tremor	2/4 cases (50%)	11/43 cases (25.6%)
Hyposmia	3/3 cases (100%)	4/12 cases (33.3%)

	CBS SAA Positive (n = 9)	CBS SAA Negative (n = 26)
Diagnostic criteria certainty		
Probable criteria	6/9 cases	18/26 cases
Possible criteria	3/9 cases	8/26 cases
Change in clinical diagnosis	1/4 cases → PD	1/12 cases → FND
Age at symptom onset, average (range)	63.6 (50–77) y	63.2 (45–75) y
Age at baseline visit, average (range)	68.8 (53–80) y	68.0 (48–79) y
Disease severity		
PSPRS, average (SD)	26.4 (11.4)	32.3 (12.5)
MDS‐UPDRS III, average (SD)	44.0 (19.8)	43.8 (13.7)
SEADL^a^	40/100 (30–90)	50/100 (20–90)
MoCA, average (SD)	16.3 (6.7)	20.2 (5.3)
Disease duration in deceased cases, average (range)	8.4 (3.0–15.2) y	8.7 (4.9–13.3) y
Disease duration in alive cases, average (range)	9.7 (9.0–10.4) y	7.3 (2.1–13.2) y
AD biomarker status		
CBS‐AD	6	7
CBS‐non‐AD	3	13
CBS‐unknown		6
Primary pathological diagnosis	AD (n = 1)	PSP (n = 2); CBD (n = 2)
LB copathology, Braak stage	1/1 case, Braak stage 1	1/4 cases, Braak stage 1
Postural light‐headedness	3/5 cases (60%)	8/18 cases (44.4%)
Visual hallucinations	0/5 cases (0%)	1/24 cases (4.2%)
Tremor	3/5 cases (60%)	10/18 cases (55.6%)
Hyposmia	1/2 cases (50%)	4/8 cases (50%)

^a^ SEADL values are presented as median (range). All SAA‐positive vs. SAA‐negative subgroup comparisons of continuous variables within the PSP and CBS groups did not reach statistical significance (*P* > 0.05) using binary logistic regression that adjusted for sex, age, and disease duration at assessment.

Abbreviations: PD, Parkinson's disease; SAA, seed amplification assay; PSP, progressive supranuclear palsy; CBS, corticobasal syndrome; PD, Parkinson's disease; PSP‐RS, PSP‐Richardson's syndrome; PSP‐SL, PSP‐speech and language disorder; PSP‐P, PSP‐parkinsonism; PSP‐PGF, PSP‐progressive gait freezing; PSP/CBS, PSP/CBS overlap; PSP‐F, PSP‐frontal; IDT, indeterminate; PSPRS, PSP rating scale; SD, standard deviation; MDS‐UPDRS III, Movement Disorder Society–Unified Parkinson's Disease Rating Scale Part III; SEADL, Schwab and England activities of daily living scale; MoCA, Montreal Cognitive Assessment; CBD, corticobasal degeneration; LB, Lewy body; FND, functional neurological disorder; AD, Alzheimer's disease; CBS‐AD, corticobasal syndrome‐Alzheimer's; CBS‐non‐AD, corticobasal syndrome‐non‐Alzheimer's.

## Discussion

In this study, we show α‐synuclein SAA positivity in people with a clinical diagnosis of PSP and CBS, and consider the clinicopathological implications of this.

In PSP, as seen in previous studies,^2^ we confirm diagnostic consistency over time and high rates of primary 4RT pathology at postmortem, such that PD‐type SAA positivity is highly unlikely to represent cases with primary PD pathology. Our PSP group rate of 5 of 59 (8.5%) PD‐type SAA positivity is comparable with the rates of Lewy body copathology seen in postmortem studies of PSP^13^, ^14^ and the MD‐GAP postmortem study, which found Lewy body copathology in 43 of 740 (5.8%) PSP cases (unpublished data). Conversely, a recent study found PD‐type SAA positivity in 4 of 16 (25%) PSP CSF samples,^15^ similar to the Lewy body copathology rate (22%) seen in a smaller postmortem PSP cohort.^16^

Our data also suggest that PD copathology may be more common in older PSP patients, suggesting that age‐related impairment in proteostasis leads to the accumulation of multiple copathologies.^16^ In addition, in contrast with previous clinicopathological studies,^13^, ^14^ our data provide preliminary evidence that PD copathology may increase disease severity and the rate of disease progression.

PD‐type SAA positivity was relatively high in our CBS group (9/37, 24.3%). Cortical Lewy body disease rarely presents with CBS, so it is unlikely that a quarter of our CBS cases had primary Lewy body pathology.^17^, ^18^ Our SAA positivity rate was higher than the rate of Lewy body copathology observed in CBD in previous neuropathology studies (10%),^16^ and in the MD‐GAP database it was 13 of 142 (9.2%) (unpublished data). It is possible that our high rate of SAA positivity may partly be explained by poor specificity of the assay in CBS‐CBD because of CBD‐type tau cross‐reacting with the α‐synuclein SAA to give false positive results, although further work is required to explore this hypothesis. This also raises the possibility that PD‐type SAA positivity in our clinically diagnosed PSP group may be because of individuals with underlying CBD pathology. Conversely, our Lewy body copathology rates may be underestimated because the sensitivity of α‐synuclein SAAs are limited in low Braak stages of Lewy body pathology,^19^ or it may be possible that the detection of α‐synuclein pathology via SAA is inhibited by the presence of tau pathology, noting that one of our pathologically diagnosed PSP participants with Braak stage 1 Lewy body copathology was PD‐type SAA negative.

Despite important conclusions, our study has several limitations. Power was low to detect small group and subgroup differences, such that the findings from our exploratory SAA‐positive versus SAA‐negative analyses are considered preliminary. There is also the potential for interrater variability impacting on our clinical measures, although significant differences across sites in this multicenter study have not been detected when analyzed previously.^20^

Although our study did not include healthy control subjects, we note that the Amprion α‐synuclein assay is a Clinical Laboratory Improvement Amendments–certified/College of American Pathologists–accredited laboratory validated assay for which performance characteristics for healthy control specimens have been established. Using an older version of the α‐synuclein SAA, which had a 150‐hour reaction time, assay positivity was detected in 478 of 545 (88%) clinically diagnosed PD cases and only 6 of 163 (4%) age‐matched healthy control subjects.^7^ Similarly, in two recent studies using the same version of the assay that we used in our study, the positivity rate in cognitively unimpaired control subjects was 5% and 16%, respectively.^21^, ^22^

We are encouraged by the fact that one of the three MSA‐type SAA‐positive cases had postmortem confirmation of primary MSA pathology; however, the method used by Amprion to differentiate between PD‐ and MSA‐type positivity is not validated.

Replication of our findings in larger cohorts with matched postmortem data will be important, together with combination testing of α‐synuclein and 4RT SAAs across parkinsonian disorders to assess positive and negative predictive values of both assays in clinical and research settings.

## Author Roles

D.P.V., R.F., M.T.J., M.H., D.L., R.O., A.M., P.N.L., K.P.B., B.C.P.G., A.C., C.K., M.T.M.H., J.B.R., H.R.M., and E.J. carried out PROSPECT‐UK study assessments. O.A., K.S.J.A., T.T.W., and Z.J. carried out postmortem assessments. D.P.V., R.F., M.T.J., M.H., T.G., L.W., J.L., C.B., and E.J. contributed to the acquisition and analysis of data. E.J. conceptualized and supervised the study. D.P.V. and E.J. drafted the manuscript. All authors critically reviewed the manuscript. E.J. takes responsibility for the integrity of the data and the accuracy of the data analysis.

## Financial Disclosures of All Authors (For the Preceding 12 Months)

Dr. Lamoureux is an employee of Amprion Inc. Dr Ghosh's salary is paid by University Hospital Southampton; in the last 12 months, he has had honoraria from the neurology masterclass and has received grants from the PSP Association; and he serves as a trustee and in the research committee for the PSP Association. Dr. Kobylecki has received speaker's honoraria from Britannia Pharmaceuticals and financial support for travel from Bial Pharma. Prof. Rowe is employed by Cambridge University with academic grants from AZ, Lilly, GSK, and Janssen, and has received paid consultancy from Asceneuron, Alector, Astex, Astronautx, Curasen, Clinical Ink, CumulusNeuro, Eisai, SV Health, Invicro, and Prevail in the last 12 months unrelated to the current work. Prof. Morris is employed by UCL; in the last 12 months he reports paid consultancy from Roche, Aprinoia, AI Therapeutics, and Amylyx; has received lecture fees/honoraria from BMJ, Kyowa Kirin, and Movement Disorders Society; and is a coapplicant on a patent application related to C9ORF72—Method for diagnosing a neurodegenerative disease (PCT/GB2012/052140).

## Supporting information


**Data S1.** Supporting Information.

## Data Availability

All data used in this study will be released via GP2 (gp2.org) and can be accessed by request to the corresponding authors.
