# Supplementary material for: Evaluation of Cerebrospinal Fluid α‐Synuclein Seed Amplification Assay in Progressive Supranuclear Palsy and Corticobasal Syndrome
Source: Mov Disord. 2024 Sep 20;39(12):2285–91. doi: 10.1002/mds.30019 (PMC11657022; doi:10.1002/mds.30019)
Supplement: Supplementary file 1 — Data S1. Supporting Information. [file MDS-39-2285-s001.docx]

**Evaluation of cerebrospinal fluid alpha-synuclein seed amplification assay in PSP and CBS**

**Supplementary information**

**Participant recruitment and diagnosis**

People with PSP and CBS who were recruited to the UK-wide PROSPECT study natural history and longitudinal cohorts between September 1, 2015 to November 1, 2023 were studied (Queen Square Research Ethics Committee 14/LO/1575) **(1)**. We obtained written informed consent from all participants who were also offered the opportunity to register for post-mortem brain donation at 1 of 4 UK brain banks (Queen Square [London], Cambridge, Oxford, and Manchester).

Participants were either retrospectively or prospectively assigned a clinical diagnosis (+/- associated subtype) in line with the 2017 MDS-PSP diagnostic criteria and 2013 Armstrong CBS diagnostic criteria **(2) (3)**, achieving “possible” or “probable” criteria. Of note, participants who fulfilled criteria for a PSP/CBS overlap syndrome were included in the PSP group for analysis.

**Clinical data collection**

At the baseline study visit we obtained clinical data including sex, age at symptom onset, age and disease duration at baseline assessment. At each study visit, we obtained scores for the PSP Rating Scale (PSPRS), the MDS Unified Parkinson’s Disease Rating Scale (MDS-UPDRS), the Montreal Cognitive Assessment (MoCA), and the Schwab and England Activities of Daily Living Scale (SEADL).

Alongside the core clinical data collection listed above, we also collected data on: 1) change in clinical diagnosis since the baseline assessment; 2) the presence or absence of hyposmia (defined by a “Sniffin’ Sticks” test score < 11 out of 16), and the presence or absence of tremor (question 2.10), postural lightheadedness (question 1.12) and visual hallucinations (question 1.2) from the MDS-UPDRS parts I and II as a binary variable, i.e. any score above 0 is defined as present, as these features are frequently encountered in synucleinopathies (PD, MSA and DLB) but are rarely seen in 4-repeat tauopathies. A comprehensive assessment of autonomic dysfunction was not carried out.

Mortality data was censored on January 1, 2024 and in deceased cases a total disease duration from symptom onset to death was calculated.

**CSF and PET imaging biomarkers of AD pathology**

CSF samples from 29 CBS cases were tested for total tau (T-tau) and Aβ1-42 levels (INNOTEST ELISA – Fujirebio Europe N.V., Gent, Belgium). CBS cases were stratified into groups with likely underlying AD pathologic features (CBS-AD), defined as cases with a CSF T-tau:Aβ1-42 ratio of greater than 1; and non-Alzheimer’s pathology (CBS-non-AD), defined as cases with a CSF T-tau:Aβ1-42 ratio of less than 1.

Two CBS cases underwent ^11^C-PiB PET Amyloid imaging using Pittsburgh Compound B (^11^C-PiB) following the protocol given in Holland et al **(4)**. ^11^C-PiB cortical standardized uptake value ratio (SUVR; 50–70 min post injection) was calculated using the whole cerebellum reference tissue as per the Centiloid Project methodology **(5)**. A negative amyloid status was characterized by a cortical ^11^C-PiB SUVR < 1.21 obtained by converting the Centiloid cut-off of 19 to SUVR using the Centiloid-to-SUVR transformation in Jack et al **(6)**.

We were unable to obtain CSF or PET imaging biomarkers of AD pathology in six CBS cases and so these were classified as CBS-unknown.

**CSF alpha-synuclein SAA analysis**

80/96 participants underwent baseline clinical assessment and lumbar puncture on the same day whilst the remaining participants had their lumbar puncture within three months of clinical assessment.

The alpha-synuclein seed amplification assay was performed in the Amprion Clinical Laboratory (CLIA ID No. 05D2209417; CAP No. 8168002) using a method validated for clinical use in accordance with Clinical Laboratory Improvement Amendment (CLIA) requirements. Each sample is analysed in triplicate in a 96-well plate using a reaction mixture comprised of 100mM PIPES pH 6.5, 0.5M NaCl, 0.1% sarkosyl, 10µM ThT, 0.3mg/mL recombinant alpha-synuclein, and 40µL CSF, in a final volume of 100µL. Two silicon nitride beads are included in each well, and positive and negative assay quality control samples are included on each plate to ensure that reliable data are being generated for each run. Plates are sealed with optical adhesive film, placed into the chamber of a BMG LABTECH FLUOstar Ω Microplate Reader, and incubated at 42°C with cycles of 1 min of shaking followed by 14 minutes of rest with fluorescence measured after every shaking cycle (excitation wavelength 440 nm, emission 490 nm).

**References**

**(1**) Jabbari E, Holland N, Chelban V, et al. Diagnosis across the spectrum of progressive supranuclear palsy and corticobasal syndrome. *JAMA Neurol* 2020; 77: 377-387.

**(2)** Höglinger GU, Respondek G, Stamelou M, et al. Clinical diagnosis of progressive supranuclear palsy: The movement disorder society criteria. *Mov Disord.* 2017; 32: 853-864.

**(3)** Armstrong MJ, Litvan I, Lang AE, et al. Criteria for the diagnosis of corticobasal degeneration. *Neurology* 2013; 80: 496-503.

**(4)** Holland N, Jones PS, Savulich G, et al. Synaptic loss in primary tauopathies revealed by [^11^C]UCB-J positron emission tomography. *Mov Disord*. 2020; 35: 1834-1842.

**(5)** Klunk WE, Koeppe RA, Price JC, et al. The Centiloid project: Standardizing quantitative amyloid plaque estimation by PET. *Alzheimer’s Dement*. 2015; 11: 1-15.e154.

**(6)** Jack CR, Wiste HJ, Weigand SD, et al. Defining imaging biomarker cut points for brain aging and Alzheimer’s disease. *Alzheimer’s Dement*. 2017; 13: 205-216.
